# Zingerone attenuates sciatic nerve damage caused by sodium arsenite by inhibiting NF-κB, caspase-3, and ATF-6/CHOP pathways and activating the Akt2/FOXO1 pathway

**DOI:** 10.22038/IJBMS.2023.74088.16094

**Published:** 2024

**Authors:** Selcuk Yilmaz, Cihan Gur, Sefa Kucukler, Nurhan Akaras, Fatih Mehmet Kandemir

**Affiliations:** 1 Department of Orthopedics and Traumatology, Faculty of Medicine, Kütahya Health Sciences University, Kütahya, Turkey; 2 Department of Biochemistry, Faculty of Veterinary Medicine, Atatürk University, Erzurum, Turkey; 3 Department of Histology and Embryology, Faculty of Medicine, Aksaray University, Aksaray, Turkey; 4 Department of Medical Biochemistry, Faculty of Medicine, Aksaray University, Aksaray, Turkey

**Keywords:** Apoptosis, Endoplasmic reticulum- stress, Inflammation, Sciatic nerve, Sodium arsenite, Zingerone

## Abstract

**Objective(s):**

In the present study, the potential protective effects of zingerone (ZNG) against sciatic nerve damage caused by sodium arsenite (SA), a common environmental pollutant, were evaluated by various biochemical, molecular, and histological methods.

**Materials and Methods:**

In the study, SA and ZNG were given to 35 male Sprague Dawley rats for 14 days. At the end of the period, the sciatic nerve tissues were taken and the markers involved in oxidative stress, endoplasmic reticulum stress, inflammation, and apoptosis were analyzed.

**Results:**

The data obtained showed that SA decreased glutathione (GSH) levels and increased malondialdehyde (MDA) levels in the sciatic nerve tissue. However, it was determined that these markers approached the control group levels due to the anti-oxidant properties of ZNG. While SA triggered endoplasmic reticulum stress and apoptosis pathways, ZNG suppressed them. Moreover, SA up-regulated inflammatory markers such as nuclear factor kappa-B (NF-κB), tumor necrosis factor-alpha (TNF-α), interleukin-1-beta (IL-1β), and neuronal nitric oxide synthases (nNOS) in the sciatic nerves and caused neuro-inflammation and inhibited cell survival by suppressing serine/threonine-protein kinase 2 (Akt2) and forkhead box protein O1 (FOXO1) genes. It has also been shown histopathologically that SA causes degeneration in the sciatic nerves. In contrast, ZNG suppressed neuro-inflammation, activated Akt2/FOXO1 signaling, and repaired histological irregularities.

**Conclusion:**

In general, SA caused oxidative stress, inflammation, ER stress, and apoptosis in the sciatic nerves of rats, causing damage to the tissues, however, ZNG suppressed these pathways and protected the sciatic nerves from the destructive effect of SA.

## Introduction

The spread of industrialization brings about the release of environmental pollutants into nature and causes many living things to be affected by this. Arsenic, one of the important environmental pollutants, is dispersed into the environment through the use of pesticides, coal and processed wood burning, and mining wastes (1). More importantly, the presence of arsenic compounds in groundwater sources indicates that millions of people are exposed to these pollutants (2, 3). Various health problems, including dysfunction in the neurological, cardiovascular, reproductive, hepatic, and renal systems, may occur after arsenic exposure (3, 4). It has been documented that a single exposure to an acute toxic dose of arsenic results in clinical symptoms such as diarrhea and vomiting, resulting in death from dehydration. In cases where patients survive, it has been reported that recovery takes weeks (5) and high arsenic exposure may be associated with persistent peripheral neuropathy (6). It is estimated that the morbidity of peripheral neuropathy, which occurs with symptoms such as decreased nerve conduction velocity and numbness in the lower extremities, paresthesia, and hyperalgesia, is 30% after arsenic exposure (7). Clinical studies have reported that arsenic causes fragmentation of myelin and axons, reduction in the number of myelinated fibers and axons, and even complete degeneration of axons (8-10). Although the causal factors for arsenic-induced peripheral neuropathy remain unclear, some evidence has been presented to suggest that oxidative stress is an important stimulus. Moreover, anti-oxidants have been reported to improve nerve conduction velocity against arsenic exposure (7). Therefore, it is thought that stronger anti-oxidant compounds will contribute to the emergence of more positive results against arsenic-induced peripheral neuropathy. 

It has been reported that natural anti-oxidants can protect the nervous system and improve behavioral functions by reducing oxidative damage (11). It has been reported that ginger is neuroprotective in different models of neurological disorders, has a high ability to reach the central nervous system, and has positive safety data (12). Zingerone (ZNG) is one of the active components of ginger and is known to have high anti-oxidant and various pharmacological effects (13). ZNG is rapidly metabolized, easily crosses the blood-brain barrier, reaches good concentration in the systemic circulation, and is eliminated from the body within 6 hr after oral administration (11). On the other hand, studies investigating the efficacy of ZNG against peripheral neuropathy are very limited.

In the present study, some molecular pathways that have important roles in peripheral neuropathy in the sciatic nerve tissue after sodium arsenite (SA) was given to rats were investigated. Also, it was examined whether ZNG has suppressive properties on these molecular pathways, and histological changes in the sciatic nerve tissue were observed.

## Materials and Methods

***Characteristics of experimental animals, care conditions, and application groups***

Rats (35 male Sprague-Dawley) were obtained from the Medical Experiment Application and Research Center (Erzurum/Turkey) and no application was made for 1 week for their adaptation to the environment before the study. Before the application, the weight of the rats was between 250 and 300 g and their age was 10-12 weeks. Animals were housed in controlled ambient conditions (12-hour light/12-hour dark cycle, humidity of 45%±5%, and room temperature of 24±1^°^C). Their nutrition was provided *ad libitum* with standard pellet feed and tap water. Animal experiments comply with the ARRIVE guidelines, and they were carried out under the UK Animals (Scientific Procedures) Act, 1986 and associated guidelines, EU Directive 2010/63/EU for animal experiments, or the National Research Council’s Guide for the Care and Use of Laboratory Animals. Approval was obtained from Atatürk University Animal Experiments Local Ethics Committee for interventions to be applied to animals (Approval number: 2023/01/11). For the applications, 5 groups of 7 animals each were created. The names of the groups are control, ZNG, SA, SA+ZNG 25, and SA+ZNG 50. Doses of SA and ZNG were determined by reference to previous studies (14, 15). Animals in the control group received saline orally for 14 days. Animals in the ZNG group received ZNG at a dose of 50 mg/kg for 14 days. Animals in the SA group received SA at a dose of 10 mg/kg for 14 days. Animals in the SA+ZNG 25 and SA+ZNG 50 groups received SA at a dose of 10 mg/kg for 14 days and were given ZNG at doses of 25 or 50 mg/kg 30 min after each SA administration. On the 15th day of the study, the animals were decapitated under mild sevoflurane anesthesia and their sciatic nerves were removed. Half of the excised tissues were taken into 10% formalin for histological examinations, while the other half was used in biochemistry analyses.

***Biochemical analyzes***

Sciatic nerve tissues taken from rats were pulverized with liquid nitrogen and homogenized in 1.15% KCl. Malondialdehyde (MDA) and glutathione (GSH) analyses were performed in homogenates. Homogenates were centrifuged at 3500 RPM for 15 min for MDA analysis and 20 min at 10000 RPM for GSH analysis. Then, while MDA analysis was performed in the supernatants with the method of Placer, Cushman, and Johnson (16), GSH levels were analyzed with the method of Sedlak and Lindsay (17).

***RT-PCR analyzes***

Total RNAs were isolated from sciatic nerve tissues with QIAzol Lysis Reagent (79306; Qiagen). Concentrations of total RNAs were measured with NanoDrop (BioTek Epoch). Total RNAs were converted into double-stranded cDNAs with the iScript cDNA Synthesis Kit (Bio-Rad). Then, the relative mRNA transcript levels of the genes whose sequences are given in Table 1 were analyzed by reacting the mixture formed with cDNAs, iTaq Universal SYBR Green Supermix (BIO-RAD), and primers in the Rotor-Gene Q (Qiagen) instrument. Calculations were made using the 2^-deltadeltaCT^ method (18) and β-Actin was used as the internal control.

***Western blot analyzes***

Proteins were extracted and purified from the sciatic nerve in the same method as was performed with other tissues. Briefly, protein samples were separated on 10% sodium dodecyl sulfate-polyacrylamide gels (30 µg/lane) and then transferred to a polyvinylidene fluoride membrane (Bio-Rad). The membrane was blocked with 5% bovine serum albumin in phosphate-buffered saline with 0.1% Tween 20 (PBS-T) buffer and then incubated overnight at 4 ^°^C with primary antibodies. The blots were treated with secondary antibodies, developed using an enhanced chemiluminescence system (ECL kit; Bio-Rad, Hercules, USA), and captured on Biorad Gel Doc XR+Imaging System (Bio-Rad, Hercules, USA) for analysis. As primary antibodies, the following kits were utilized: mouse monoclonal anti-NF-κB p65 (1:500; Santa Cruz, CA), mouse monoclonal anti-nNOS (1:500; Santa Cruz, CA), mouse monoclonal anti-IL-1β (1:500; Santa Cruz, CA), and mouse monoclonal anti- β-Actin (1:500; Santa Cruz, CA). The secondary antibody was an anti-mouse antibody that was conjugated with HRP (1:2000; Santa Cruz, CA).

***Histopathological analyzes***

At the end of the experiment, the sciatic nerves removed from the rats were kept in 10% formalin solution for 48 hr to be fixed. Then, the fixed tissues were dehydrated by passing through a series of increasing grades of alcohol, transparent with xylol, and finally embedded in paraffin. Sections of 4 µm thickness were taken from the paraffin-blocked sciatic nerves using microton and hematoxylin-eosin (H&E) staining was performed. Stained slides were examined and photographed using a light microscope (Olympus Cx 43; Japan).

***Statistical analyzes***

One-way analysis of variance (ANOVA) and Tukey comparison tests were used in the statistical analysis of the data. 

## Results

***MDA and GSH levels in sciatic nerve tissue***

**Figure 1:**
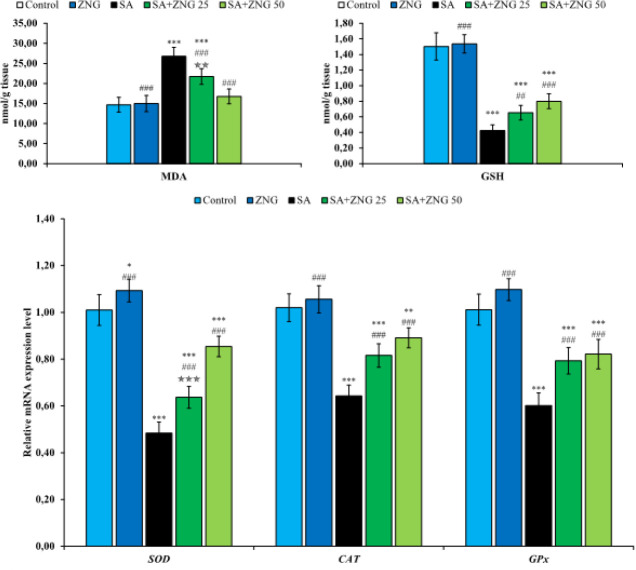
Effects of sodium arsenite and zingerone administrations on MDA and GSH levels and SOD, CAT, and GPx gene expression levels in sciatic nerve tissues of rats Statistical significance: Control vs Others; ***P<0.001, **P<0.01, *P<0.05, SA vs Others; ^###^*P<*0.001, ^##^*P<*0.01, ^#^*P<*0.05, SA+ZNG 25 vs SA+ZNG 50; ^✯✯✯^*P<*0.001, ^✯✯^*P<*0.01, ^✯^*P<*0.05

MDA and GSH levels in sciatic nerve tissue are presented in Figure 1. The data obtained show that MDA levels increased in the sciatic nerves of rats given SA. Moreover, after SA application, it is seen that there is a decrease in GSH stores in the tissue. ZNG treatment, on the other hand, reduced MDA levels in a dose-dependent manner. GSH levels increased after ZNG treatment, but there was no statistical difference between doses.

***Relative mRNA transcript levels of SOD, CAT, and GPx enzymes in sciatic nerve tissue***

To determine the effects of SA and ZNG administrations on oxidative stress in sciatic nerves, the relative mRNA transcript levels of anti-oxidant enzymes superoxide dismutase (SOD), catalase (CAT), and glutathione peroxidase (GPx) in the tissue were analyzed and the results are presented in Figure 1. While SA application was observed to suppress SOD, CAT and GPx genes in sciatic nerves, it was determined that the expressions of these genes were up-regulated after ZNG treatment compared to the SA group. It was determined that a high dose was more effective on the SOD gene, however, there was no significant difference between a high dose and a low dose in CAT and GPx genes.

***Relative mRNA transcript levels of ER stress markers in sciatic nerve tissue***

**Figure 2:**
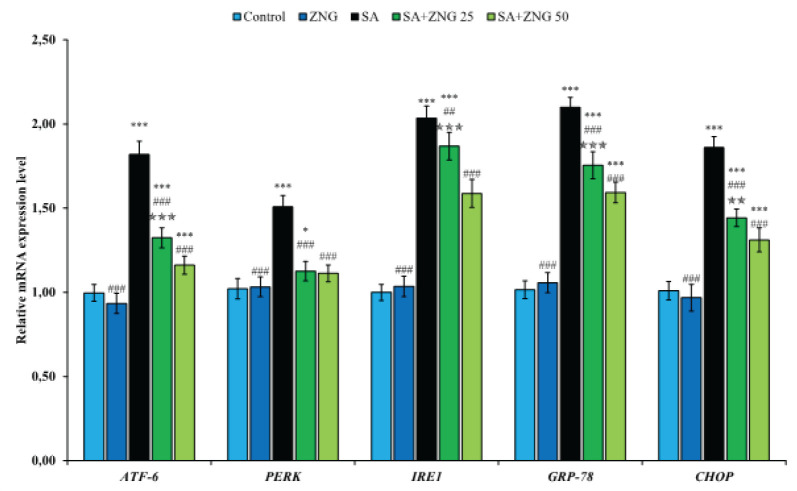
Effects of sodium arsenide and zingerone administrations on ATF-6, PERK, IRE1, GRP-78, and CHOP gene expression levels in sciatic nerve tissues of rats Statistical significance: Control vs Others; ***P<0.001, **P<0.01, *P<0.05, SA vs Others; ^###^*P<*0.001, ^##^*P<*0.01, ^#^*P<*0.05, SA+ZNG 25 vs SA+ZNG 50; ^✯✯✯^*P<*0.001, ^✯✯^*P<*0.01, ^✯^*P<*0.05

Expressions of activating transcription factor-6 (ATF-6), double-stranded RNA-activated protein kinase (PKR)-like ER kinase (PERK), inositol-requiring enzyme-1 (IRE1), glucose-regulated protein 78 (GRP-78), and CCAAT/enhancer binding proteins (C/EBP) homologous protein (CHOP) genes were analyzed to determine ER stress in sciatic nerve tissue. According to the data presented in Figure 2, SA application caused ER stress in sciatic nerves and triggered the expression of related genes. However, ZNG treatment suppressed ER stress and down-regulated ATF-6, PERK, IRE1, GRP-78, and CHOP genes. Considering the relationship between the doses, it was determined that the high dose was more effective, except for the PERK gene. 

***Relative mRNA transcript and protein levels of markers of inflammation in sciatic nerve tissue***

**Figure 3:**
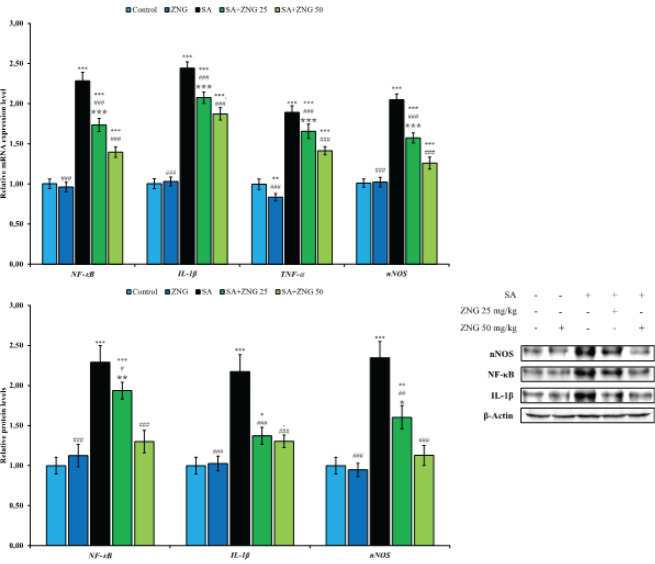
Effects of sodium arsenide and zingerone administrations on NF-κ, IL-1β, TNF-α, and nNOS gene expression levels and NF-κ, IL-1β, and nNOS relative protein levels in sciatic nerve tissues of rats Statistical significance: Control vs Others; ***P<0.001, **P<0.01, *P<0.05, SA vs Others; ^###^*P<*0.001, ^##^*P<*0.01, ^#^*P<*0.05, SA+ZNG 25 vs SA+ZNG 50; ^✯✯✯^P<0.001, ^✯✯^P<0.01, ^✯^P<0.05

To determine the effect of SA and ZNG applications on inflammation in the sciatic nerve tissue, nuclear factor kappa-B (NF-κB), tumor necrosis factor-alpha (TNF-α), interleukin-1-beta (IL-1β), and neuronal nitric oxide synthases (nNOS) genes were analyzed by RT-PCR method, and NF-κB, IL-1β and nNOS proteins were analyzed by the western blot method. According to RT-PCR results, it was observed that SA administration could trigger inflammation by up-regulating NF-κB, TNF-α, IL-1β, and nNOS genes, however, ZNG treatment could suppress these genes in a dose-dependent manner. Moreover, a significant increase in NF-κB, IL-1β, and nNOS protein levels was detected with SA administration. While it was observed that ZNG treatment significantly reduced the levels of these proteins, it was determined that there was no difference between doses on IL-1β levels. All results are summarized in Figure 3.

***Relative mRNA transcript levels of apoptosis markers in sciatic nerve tissue***

**Figure 4:**
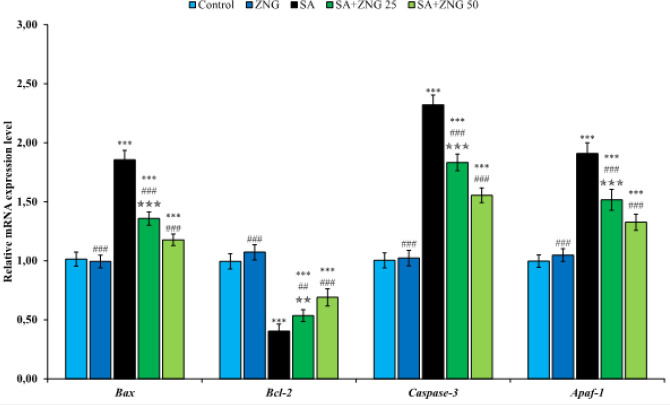
Effects of sodium arsenide and zingerone administrations on Bax, Bcl-2, Caspase-3, and Apaf-1 gene expression levels in sciatic nerve tissues of rats Statistical significance: Control vs Others; ***P<0.001, **P<0.01, *P<0.05, SA vs Others; ^###^*P<*0.001, ^##^*P<*0.01, ^#^*P<*0.05, SA+ZNG 25 vs SA+ZNG 50; ^✯✯✯^*P<*0.001, ^✯✯^*P<*0.01, ^✯^*P<*0.05

Apoptosis status in sciatic nerve tissue was evaluated by analyzing mRNA transcript levels of Bcl-2-associated X protein (bax), B-cell lymphoma 2 (bcl-2), caspase-3, and Apoptotic protease activating factor 1 (apaf-1) genes. According to the results presented in Figure 4, SA triggered the bax, caspase-3, and apaf-1 genes and suppressed the bcl-2 gene in the sciatic nerves. Thus, it was determined that it may cause apoptosis in the sciatic nerve tissue. ZNG, on the other hand, suppressed apoptotic genes in a dose-dependent manner, while up-regulating the anti-apoptotic gene.

***Relative mRNA transcript levels of Akt2 and FOXO1 in sciatic nerve tissue***

**Figure 5:**
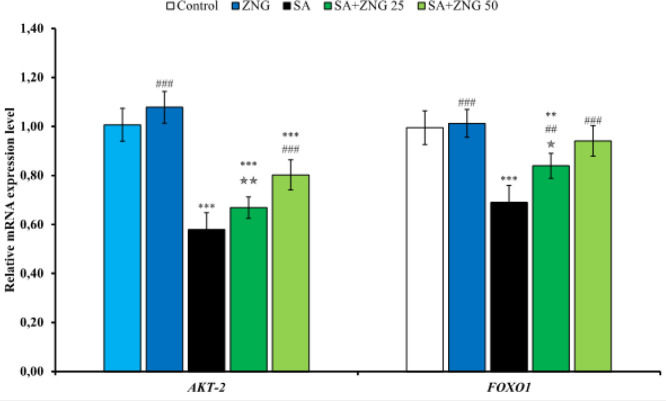
Effects of sodium arsenide and zingerone administrations on Akt-2 and FOXO1 gene expression levels in sciatic nerve tissues of rats Statistical significance: Control vs Others; ***P<0.001, **P<0.01, *P<0.05, SA vs Others; ^###^*P<*0.001, ^##^*P<*0.01, ^#^*P<*0.05, SA+ZNG 25 vs SA+ZNG 50; ^✯✯✯^*P<*0.001, ^✯✯^*P<*0.01, ^✯^*P<*0.05

The mRNA transcript levels of the serine/threonine-protein kinase 2 (Akt2) and forkhead box protein O1 (FOXO1) genes were also analyzed in the sciatic nerve tissue. The data obtained (Figure 5) showed that SA administration suppressed these genes, and these genes were triggered after ZNG was given to the animals. It has been observed that giving 50 mg/kg ZNG is more effective than 25 mg/kg.

***Histopathological evaluation results***

**Figure 6:**
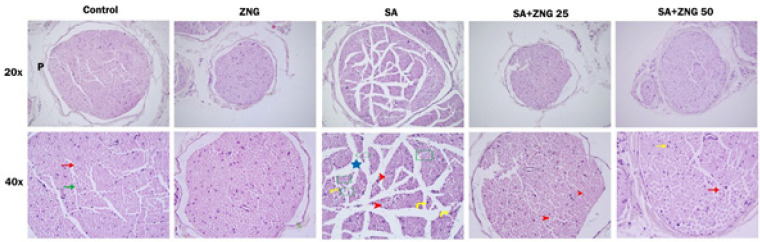
Histological photographs of the sciatic nerves of rats (H&E, original magnification; top row 20x; bottom row 40x)

According to the H&E staining results, the sciatic nerve fascicles and fibers belonging to the control and ZNG groups were in regular alignment and well-organized, the perineurium was normal in thickness, and there was no vacuolization and edema. Histopathological changes were observed when the sciatic nerves of the SA group were examined. In particular, nerve fibers were irregular, vacuolization, axonal shrinkage, atrophy, and axonal demyelination were observed in some axons. Especially in this group, the gaps between the fibers increased. As a result of the administration of ZNG together with SA, the alignment of the fibers forming the nerve fascicles showed an organization close to control. With the administration of ZNG, a decrease in vacuolization and an increase in Schwann cell activation were observed. Overall, HE staining showed that ZNG treatment promoted recovery of nerve histological changes (Figure 6).

**Table 1.** Sequences of primers of genes used in RT-PCR method

**Gene**	**Sequences (5’-3’)**	**Length (bp)**	**Accession No**
**SOD**	F: AATGTGGCTGCTGGAAAGGA R: GCTTCCAGCATTTCCAGTCT	171	NM_017050.1
**CAT**	F: CTGAGAGAGTGGTACATGCA R: AATCGGACGGCAATAGGAGT	130	NM_012520.2
**GPx**	F: CAAGGTGCTGCTCATTGAGA R: ATGTCCGAACTGATTGCACG	139	NM_030826.4
**NF-** **B**	F: AGTCCCGCCCCTTCTAAAAC R: CAATGGCCTCTGTGTAGCCC	106	NM_001276711.1
**IL-1**	F: ATGGCAACTGTCCCTGAACT R: AGTGACACTGCCTTCCTGAA	197	NM_031512.2
**TNF-**	F: CTCGAGTGACAAGCCCGTAG R: ATCTGCTGGTACCACCAGTT	139	NM_012675.3
**nNOS**	F: TGGAGACATCATTCTCGCAG R: GATGTGTAGTGAAGCCCTCA	140	NM_052799.2
**Akt2**	F: GAGTACTTGCACTCGACGGA R: CCATGAGGATGAGCTCGAAG	304	NM_017093.1
**FOXO1**	F: CAGCCAGGCACCTCATAACA R: TCAAGCGGTTCATGGCAGAT	143	NM_001191846.3
**ATF-6**	F: TCAACTCAGCACGTTCCTGA R: GACCAGTGACAGGCTTCTCT	130	NM_001107196.1
**PERK**	F: GATGCCGAGAATCATGGGAA R: AGATTCGAGAAGGGACTCCA	198	NM_031599.2
**IRE1**	F: GCAGTTCCAGTACATTGCCATTG R: CAGGTCTCTGTGAACAATGTTGA	163	NM_001191926.1
**GRP78**	F: CATGCAGTTGTGACTGTACCAG R: CTCTTATCCAGGCCATATGCAA	143	NM_013083.2
**CHOP**	F: GAAGCCTGGTATGAGGATCT R: GAACTCTGACTGGAATCTGG	209	NM_001109986.1
**Caspase-3**	F: ACTGGAATGTCAGCTCGCAA R: GCAGTAGTCGCCTCTGAAGA	270	NM_012922.2
**Bax**	F: TTTCATCCAGGATCGAGCAG R: AATCATCCTCTGCAGCTCCA	154	NM_017059.2
**Bcl-2**	F: GACTTTGCAGAGATGTCCAG R: TCAGGTACTCAGTCATCCAC	214	NM_016993.2
**Apaf-1**	F: ACCTGAGGTGTCAGGACC R: CCGTCGAGCATGAGCCAA	192	NM_023979.2
**-Actin**	F: CAGCCTTCCTTCTTGGGTATG R: AGCTCAGTAACAGTCCGCCT	360	NM_031144.3

MDA: Malondialdehyde, GSH: Glutathione, SOD: Superoxide dismutase, CAT: Catalase, GPx: Glutathione peroxidase, SA: Sodium arsenite, ZNG: Zingerone

ATF-6: Active transcription factor-6, IRE1: Inositol-requiring enzyme-1, GRP-78: Glucose-regulated protein 78, CHOP: C/EBP homologous protein, SA: Sodium arsenite, ZNG: Zingerone

NF-κ: Nuclear factor kappa-B , IL-1β: Interleukin-1-beta, TNF-α: Tumor necrosis factor-alpha, nNOS: Neuronal nitric oxide synthases, SA: Sodium arsenite, ZNG: Zingerone

Bax: Bcl-2-associated X protein, Bcl-2: B-cell lymphoma 2, Apaf-1: Apoptotic protease activating factor 1, SA: Sodium arsenite, ZNG: Zingerone

Akt-2: Serine/threonine-protein kinase 2, FOXO1: forkhead box protein O1, SA: Sodium arsenite, ZNG: Zingerone

Control group: The group containing normal nerve axons (red arrow) and myelin sheathed (green arrow) axons surrounded by thin perineurium (P), ZNG (Zingeron) group, SA (sodium arsenide) group: Vacuolization in axons (arrowhead), loss of myelin (circle), shrinkage of axonal myelin (square), severe separation of sciatic fibers (star), nuclei of different sizes of Schwann cells and fibroblasts (curved arrow) are seen. SA+ZNG 25 (Zingerone+ Sodium Arsenite 25) group: nerve fibers show slight vacuolization (arrowhead) and smooth organization. SA+ZNG 50 (Zingerone+ Sodium Arsenite 50) group: it contains increased Schwann cell activation (yellow arrow) and normal axon (red arrow)

## Discussion

In the present study, the negative effects of SA, which is an important environmental pollutant, on the sciatic nerve tissue of rats were examinedm and it was investigated whether ZNG had a protective effect against these negative effects.

Peripheral neuropathy is a peripheral nervous system disorder accompanied by symptoms such as pain, abnormal temperature perception, numbness, weakness, and muscle atrophy caused by damage to peripheral nerves. Increasing evidence suggests that oxidative stress plays an important role in the development of peripheral neuropathy (19). Since peripheral nerves do not have a vascular barrier, they are vulnerable to attack by toxic agents and their structures can accumulate neurotoxic substances. Moreover, peripheral nerves are known to have weak cellular anti-oxidant defense mechanisms (20, 21). Several studies have focused on the role of arsenic-induced oxidative stress in peripheral neurotoxicity. In the dorsal root ganglion (DRG), SA has been reported to cause the depletion of GSH stores, which play a vital role in cellular anti-oxidant defense (22, 23). In the current study, it was observed that GSH stores were depleted in the sciatic nerve tissue of rats given SA, and in addition, MDA levels, which is a strong indicator of lipid peroxidation and therefore oxidative stress (24, 25), increased. SOD, CAT, and GPx enzymes are another component of cellular anti-oxidant defense and play an active role in scavenging radicals such as superoxide anion and hydrogen peroxide that cause serious damage to cells (26-28). However, various toxic compounds cause toxicities by suppressing the effectiveness of these anti-oxidants (29-31). In the present study, these enzymes were analyzed at the gene level and it was determined that SA significantly suppressed these genes. When evaluated together, it has been observed that SA may cause peripheral neuropathy by triggering oxidative stress in the sciatic nerve tissue. On the other hand, ZNG, which attracts attention with its anti-oxidant feature, probably scavenges the free radicals originating from SA and renews its GSH stores. Also, by up-regulating SOD, CAT, and GPx genes, it strengthened anti-oxidant defense and reduced lipid peroxidation in sciatic nerves.

The axonal endoplasmic reticulum causes misfolded proteins on ribosomes under stress, and misfolded proteins begin to accumulate in the ER lumen. The accumulation of these proteins is sensed by transmembrane sensors (PERK, IRE1, and ATF-6) located in the ER and triggers the unfolded protein response to restore cellular homeostasis (32). However, prolongation of the UPR promotes cellular apoptosis and necrosis (33). It does this by first activating c-jun N-terminal kinase (JNK) and p38 MAPK, and then activation of pro-apoptotic genes belonging to the Bcl-2 family of these factors, cytochrome c release, and suppression of Bcl-2 (34, 35). In addition, ER stress contributes to apoptosis by activating the caspase pathway independently of cytochrome c (32). Many toxic agents trigger ERS and cause peripheral neuropathy (33, 36, 37). There is also evidence that arsenic also activates ERS (38, 39). In our study, it was determined that SA up-regulated the indicators of ER stress (ATF-6, PERK, IRE1, GRP-78, and CHOP) in the sciatic nerve tissue. On the other hand, the expression of these sensors was suppressed by ZNG treatment. The ability of ZNG to inhibit ERS is thought to be due to its anti-oxidant activity. Because recent evidence shows a close relationship between ERS and oxidative stress (40, 41). Neuronal apoptosis plays a critical role in the development of neuropathic pain, and inhibition of neuronal apoptosis in nerve injury models has been reported to prevent pain-related behavior (42). Considering that oxidative stress triggers ER stress and ER stress triggers apoptosis, it is thought that anti-oxidant compounds will protect against the development of peripheral neuropathy by suppressing neuronal apoptosis. Based on this hypothesis, in the study, ZNG was given to rats after SA administration and it was observed that apoptotic genes (Bax, Apaf-1, and Caspase-3) triggered by SA were down-regulated after ZNG treatment. In addition, Bcl-2, the anti-apoptotic gene (43) suppressed by SA, was triggered after ZNG treatment. Studies investigating the effects of SA on apoptosis in sciatic nerve tissue are limited. However, many studies are showing that SA triggers apoptosis by activating Bax, caspase-3, and apaf-1 genes and suppressing bcl-2 in various tissues (44, 45).

Akt signaling is involved in the regulation of various cellular functions such as transcriptional regulation, angiogenesis, proliferation, apoptosis, and survival. It is known that the Akt signal has an important role in pain behavior (46). Akt exerts an anti-apoptotic effect by regulating the expression of Bcl-2 family members and caspase-3 and contributes to cell survival (47-49). Previous studies have shown that Akt signaling prevents neuronal cell death by activating neurotrophic factors (50, 51). FOXO1, a transcription factor of the Forkhead Box O subfamily (FOXO), is also involved in a variety of important cellular processes, including cellular differentiation, apoptosis, and cell cycle progression, similar to Akt, and there is a close relationship between Akt signaling and FOXO1 (52). In the present study, it was determined that SA inhibited Akt2 and FOXO1 signals in sciatic nerves and had a negative effect on cell survival. On the other hand, it was observed that the Akt2/FOXO1 pathway was activated after ZNG treatment, and sciatic nerve cells were protected from the toxic effect of SA.

Various toxic drugs induce painful peripheral neuropathy by inducing proinflammatory cytokines, nitric oxide, prostaglandins, etc. These mediators are downstream signals of a transcription factor, NF-κB (53). NF-κB is a pleiotropic modulator that migrates from the cytosol to the nucleus after dissociation from IκB and regulates the expression of up to 500 different genes in the nucleus, including genes that replicate proinflammatory cytokines such as TNF-α and IL-1β (53, 54). Previous studies have shown that compounds with anti-oxidant properties can provide protection against peripheral neuropathy by attenuating neuro-inflammation (20, 47). In our study, it has been shown that SA triggers the expression of inflammatory markers such as TNF-α, IL-1β, and nNOS together with NF-κB, causing neuro-inflammation and this may result in sciatic nerve damage. On the other hand, these inflammatory markers were suppressed and sciatic nerve damage was alleviated with ZNG treatment.

## Conclusion

The data obtained showed that SA caused sciatic nerve damage by triggering oxidative stress, inflammation, and apoptosis, however, these pathways were suppressed after ZNG treatment, and the damage to the sciatic nerves was alleviated.

## Authors’ Contributions

S Y provided conceptualization, methodology, formal analysis, and investigation. C G contributed to writing, review, editing, data curation, and methodology. S K provided data curation, methodology, and conceptualization. N A and FM K contributed to data curation and methodology.

## Conflicts of Interest

The authors declare no conflicts of interest.
